# What motivates medical students to select medical studies: a systematic literature review

**DOI:** 10.1186/s12909-018-1123-4

**Published:** 2018-01-17

**Authors:** Sonu Goel, Federica Angeli, Nonita Dhirar, Neetu Singla, Dirk Ruwaard

**Affiliations:** 1 School of Public Health, PGIMER, Sector-12, Chandigarh, 160012 India; 2 Department of Health Services Research, Care and Public Health Research Institute, Faculty of Health, Medicine and Life Sciences, Maastricht University, Maastricht, The Netherlands; 3 Department of Organization Studies, School of Social and Behavioural Sciences, Tilburg University, Tilburg, the Netherlands; 4 Department of Health Services Research, Care and Public Health Research Institute, Faculty of Health, Medicine and Life Sciences, Maastricht University, Maastricht, The Netherlands

**Keywords:** Motivation, Medical students, Rural areas, Systematic review

## Abstract

**Background:**

There is a significant shortage of health workers across and within countries. It is of utmost importance to determine the factors that motivate students to opt for medical studies. The objective of this study is to group and review all the studies that investigated the motivational factors that underpin students’ selection of medical study in recent years.

**Methods:**

The literature search was carried out by two researchers independently in PubMed, Google Scholar, Wiley and IndMED databases for articles published from year 2006 till 2016. A total of 38 combinations of MeSH words were used for search purpose. Studies related to medical students and interns have been included. The application of inclusion and exclusion criteria and PRISMA guidelines for reporting systematic review led to the final selection of 24 articles.

**Results:**

The majority of the studies (*n* = 16; 66.6%) were from high-income countries followed by an equal number from upper-middle and lower-middle income countries (*n* = 4,16.7%). None of the studies were from low-income countries. All of the studies were cross-sectional in nature. The main motivating factors that emerged were scientific (interest in science / medicine, social interest and academia, flexible work hours and work independence), societal (prestige, job security, financial security) and humanitarian (serving the poor and under priviledged) in high-, upper-middle and lower-middle income countries, respectively. The findings were comparable to Maslow’s hierarchy of needs theory of motivation.

**Conclusion:**

This systematic review identifies the motivational factors influencing students to join medical studies in different parts of the globe. These factors vary per country depending on the level of income. This study offers cues to policy makers and educators to formulate policy in order to tackle the shortage of health workers, i.e. medical doctors. However, more research is needed to translate health policy into concrete and effective measures.

## Background

The world is currently facing a dual problem of shortage and inequitable distribution of health workers, especially in middle- and low-income countries [1]. The World Health Organization (WHO) estimated a need for an additional 4.3 million health workers in 57 countries to fulfill the Millennium Development Goals [2]. In addition, 83 countries (44.6%) do not currently meet the 2006 World Health Report threshold of 22.8 skilled health professionals per 10,000 population [3]. Among many, the main reasons cited for shortage of health workers in rural areas include poor working conditions, lack of accommodation, lack of transport, poor pay structure, overburden with additional administrative responsibility and political interference [4]. In middle- and low-income countries, the situation is more critical because of migration of doctors to high-income (developed) countries whereas inequitable distribution of health workers between urban and rural areas is primarily due to poor motivation of health workers to work in rural areas [5].

The choice of medical study depends upon various factors such as interest in the medical field, good job opportunities, a desire to serve others, medical background of the parents and many more [6, 7]. In literature, no review has been conducted in the last ten years about motivation factors of students to select medical studies. The existing reviews have either been conducted before ten years or with different objectives [8, 9]. One review by Puertas et al. [8] published in 2013 was conducted to review the factors influencing medical student’s choice in primary care while another one by Brissette and Howes [9] published in 2010 was conducted on the articles available till 2008. Brisstte and Howes identified that motivation to take up medical studies lies in addressing learner’s needs for competence, autonomy, and relatedness. Providing optimal challenge and positive performance feedback, choice and opportunity for self-direction, and a sense of belongingness and connection to the medical profession can all be focused on to address the above mentioned motivators [9]. The review has given points for educators to act upon.The lacunae left by the previous review studies need to be addressed in a finer manner in context with the current challenge of the global workforce.

In last few years, human resources for health has attracted substantial scholarly attention. Over the last decade, there have been advancement in different fields of medical sciences, from prevention, patient care to laboratory workup and management of severe diseases and palliation. With the growing population and improving health care owing to better technologies, it is gravely important to improve the medical workforce, mostly doctors.

Globally, several health-related goals and programs are giving priority to human resource development in the health sector. The major health related initiatives like Sustainable Development Goals [10] and WHO’s six building blocks [11] focus on human resource development for achieving universal health coverage. The National health programs, like the National Health Mission in India, focuses on increasing human resources to upbring the health care services in the country.

The prospective medical students form a significant pool of health care workers that can help overcome the shortage globally. Therefore, understanding the current common motivational factors is essential and a summary of the factors through a review of these studies would derive a clearer picture. A strong predictor for any student to take up a career in any field is the motivation or drive from within. Motivation is defined as the process that initiates, guides, and maintains goal-oriented behaviors. It involves the biological, emotional, social, and cognitive forces that activate behavior. Fulfillment of needs results in some type of behavior, which can be either intrinsic or extrinsic [7]. Understanding motivation is very important in the medical sector because a motivated individual is willing to exert and maintain an effort to provide good-quality health services.

The objective of this study is to group and review all the studies that investigated the motivational factors that underpin students’ selection of medical study in recent years.

## Methods

### Search strategy

The literature search was carried out with the purpose to identify the perceptions of medical students to enter medical studies. The search was carried out by two researchers (NS and ND) independently in PubMed, Google Scholar, Wiley and IndMED databases for original studies conducted from 2006 to 2016. This time frame was chosen as many studies were done during this period to identify the motivational factors. MeSH and free-text terms “(Motivat*) AND (select* OR choice OR choose) AND (medical student* OR medical school* OR interns) have been used. Internship in the period of practical application of theoretical (mostly) knowledge of the previous medical school years, hence interns were also made a part of the search strategy. Search terms and keywords were altered as per specification of individual databases. A total of 38 combinations were used for search purpose.

**Fig. 1:**
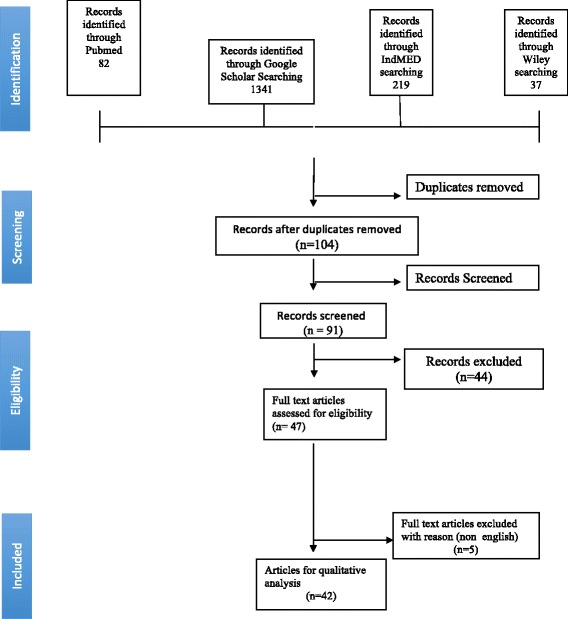
Flow chart of selection and exclusion of studies for the systematic review

An initial search identified thousands of related records from the Google scholar, PubMed, Ind Med and Wiley online library databases. The articles which were not related to motivation were excluded at the first step. Then search results were imported to Microsoft Excel and duplications were removed by sorting the titles of articles. The selected studies were then screened by reading the title and abstract resulting in shortlisting 91 articles. Of these, 62 articles were excluded based on eligibility criteria. The remaining 29 full-text articles were further assessed, and five were excluded because the articles were in Korean, Spanish and Chinese. A total of 24 studies were selected. Any differences of opinion were debated and consensus was reached. Further differences were resolved by the third researcher (SG). PRISMA guidelines were strictly followed during the study. Figure 1 represents the flow chart leading to sample selection.

### Selection criteria and sample

All studies carried out and published from year 2006 till 2016 were included in the review. Inclusion criteria were studies describing motivation to study medicine, conducted among medical students and interns and available in English language. Exclusion criteria were those studies done before 2006, published in languages other than English, and those not related to motivation or medical students and interns.

### Data analysis

A thematic analysis of selected papers was performed, wherein two research assistants coded the papers independently and reached consensus on relevant themes [12]. They also extracted details of the final articles using a standardized abstraction form that collected information on: the author, the journal, the year of publication, location, study objectives, study design, major findings, limitations, and observations. In this paper, we systematically review the literature related to medical education with the goal of identifying the motivating factors influencing the medical students to join medical studies.

The results of the studies’ review were categorized under different heads viz. scientific factors, social factors and humanitarian factors based upon criteria devised by Goel S et al. in their study on development and validation of the motivations for selection of medical study in India [13]. In this study a ‘Motivation of Selection of Medical Study (MSMS)’ tool was developed using extensive literature review followed by Delphi technique. The three domains and the issues that emerged are shown in Table 1.

**Table 1:** Domains and issues that emerge as main motivational factors

Domains	Issues
Scientific	• Ability to use new cutting edge technologies • Interest in medicine as a subject matter • Opportunities to travel and work internationally • Research opportunities • Loss of a loved one
Societal	• Job security • Social status/prestige • High income • Proposed by parents
Humanitarian	• Desire to help others • Desire to give back to their home community or country

### Ethical considerations

The study was granted ethical approval from the Institute’s Ethical Committee, PGIMER, Chandigarh (PGI/IEC/2012/810–1 P-154). Since the study is a systematic review of studies and individual level data is neither obtained nor presented, the consent.

## Results

**Fig. 2:**
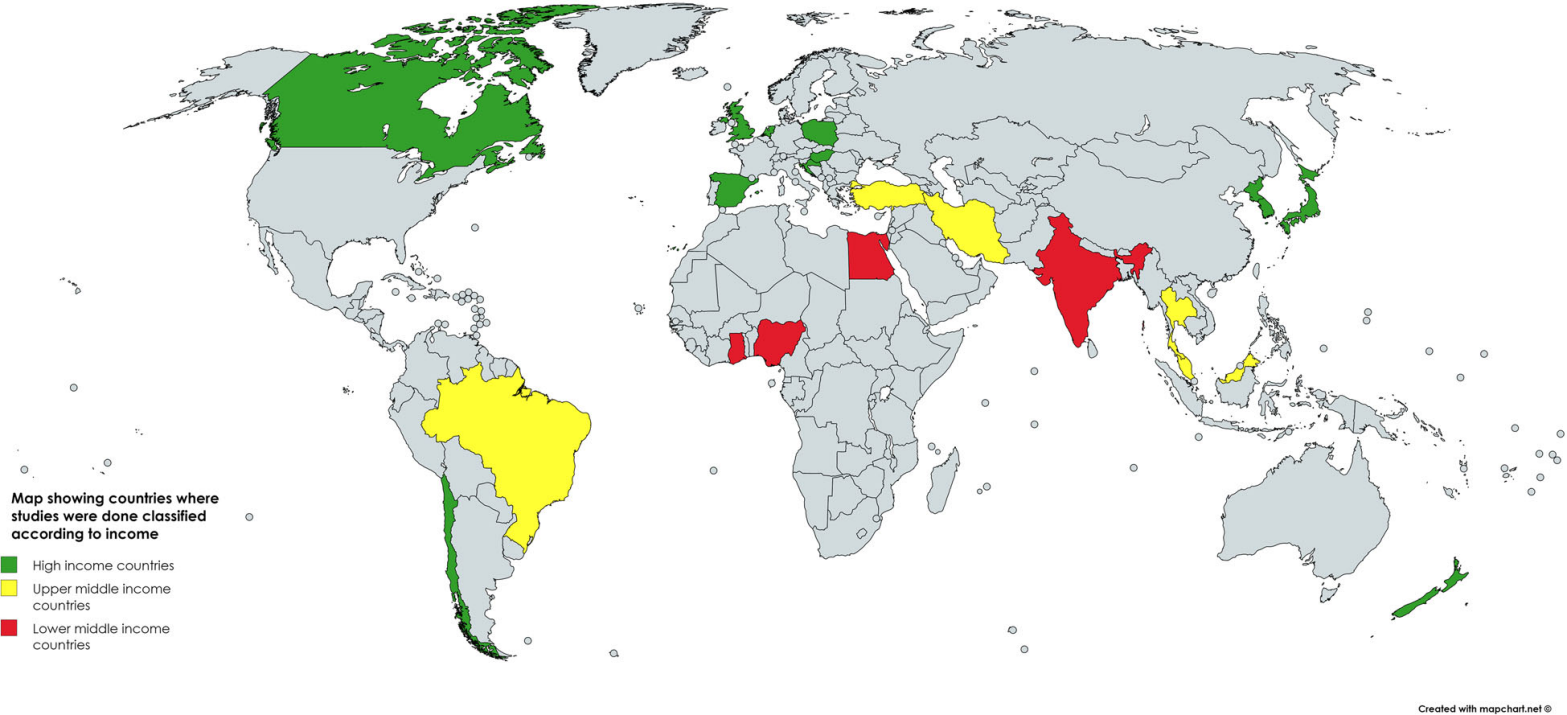
Geographical distribution of the different studies across the globe (used a web page https://mapchart.net which is free of cost and specifically designed for making customised maps)

The characteristics of the studies included in the systematic review are shown in Table 2. The assessment of factors of motivations for medical students to select medical studies was based on the World Bank categorization of low-, middle- and high-income countries [14].The low-income, lower middle-income, upper middle-income and high-income economies are defined as those with a Gross National Income (GNI) per capita of $1005 or less, between $1006 and $3955, between $3956 and $12,235 and $12,236 or more, respectively in the year 2016. The majority of the studies (*n* = 16, 66.6%) were from high-income countries followed by an equal number from upper middle and lower middle income countries (*n* = 4,16.7%). None of the studies were from low-income countries. All of the studies were cross sectional in nature (*n* = 24). Figure 2 shows the geographic distribution of the different studies.

**Table 2:** Characteristics of the studies included in the systematic review (*n* = 24)

Characteristic	Number (%)
Income Group	
• high-income countries	16 (66.6)
• upper-middle income countries	4 (16.7)
• low-income countries	4 (16.7)
Study design	
• cross sectional studies	24 (100%)
Study type	
• quantitative	21 (87.5)
• qualitative	1 (4.2)
• mixed methods	2 (8.3)

### Predominance of motivating factors according to income group

**Fig. 3:**
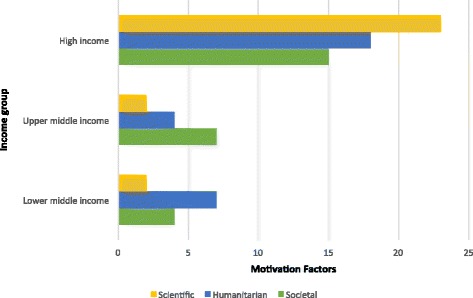
Categorization of motivation factors across different income country groups

Results reported for motivation to select medicine by medical students changes in the context of place (see Fig. 3 and Table 3). The choice of medical study among students differs between students in high-income countries, and those in upper-middle and lower–middle-income countries. The individual motivation factors that emerged are presented in Table 4.

**Table 3:** Factors affecting motivations of medical students to select medicine across different country income groups

S. No	Name of the study	Name of author	Year	Type of study	Place of study	No. of participants	Factors categorization	Individual factors
High Income Countries								
1.	Career motivation and burnout among medical students in Hungary – could altruism be a protection factor?	Gyorffy Z et al.	2016	Cross- Sectional Quantitative	Hungary	733	• Humanitarian • Scientific	• Altruistic motivation • Gaining a degree, • Finding a job, • Accessing career opportunities.
2.	Differences in medical students’ academic interest and performance across career choice motivations	Kim KJ et al.	2016	Cross –Sectional Quantitative	South Korea	195	• Scientific • Societal	• Intellectual curiosity • Personal illness / or death of family members • social prestige • financial gain
3.	Motivation towards medical career choice and future career plans of Polish medical students.	Gąsiorowski J et al.	2015	Cross- Sectional Quantitative	Poland	262	• Scientific • Societal	• Desire to help others • Interest in medical subjects
4.	Why medicine? Analyzing students’ motives for studying medicine	Becker JC et al.	2015	Cross- Sectional Quantitative	Germany	1545	• Scientific • Humanitarian	• Many-faceted workspaces • varied tasks • helping patients • scientific interest • good career prospects
5.	Studying medicine – a cross-sectional questionnaire-based analysis of the motivational factors which influence graduate and undergraduate entrants in Ireland	Sulong S et al.	2014	Cross- Sectional-Quantitative	Ireland	305	• Scientific • Societal • Humanitarian	• intellectual satisfaction, • encouragement by family/friends • financial reasons, and • professional independence.
6.	A qualitative analysis of statements on motivation of applicants for medical school	Wouters A et al.	2014	Cross- Sectional-Qualitative	Amsterdam	96	• Scientific • Humanitarian • Societal	• Research opportunities • Interest in subject • Loss of loved one • Opportunities to travel/work abroad • Contact with people
7.	Applicants to the University of Adelaide Medical School: Influences, motivation and alternative career choices	Laurence CM et al.	2013	Cross- Sectional Quantitative	Australia	1097	• Scientific • Humanitarian	• desire to help others • an affinity for science and • enjoying interacting with others
8.	Interests and perspectives of first and last year medical students	Toso A et al.	2012	Cross- Sectional Quantitative	Spain	415	• Scientific • Humanitarian	• social interest • interest in science and academia.
9.	The medical career choice motivation- results from Hungarian study	Girasek E	2011	Cross- sectional Quantitative	Hungary	560	• Scientific • Humanitarian	• General interest • Intellectual work • Helping profession • Social utility
10.	Effects of age, gender and educational background on strength of motivation for medical school	Kusurkar RA et al.	2010	Cross- sectional Quantitative	Netherland	620	• Scientific	• Higher age • Pre-entrance educational background
11.	Motivation, study habits, and expectations of medical students in Singapore	Amin Z	2009	Cross- Sectional Mixed methods	Singapore	192	• Scientific • Societal	• interesting challenging job • Family encouragement • prefer to study overseas
12.	A two factor model of performance approach goals in student motivation for starting medical school.	Wilson JI	2009	Cross- Sectional Quantitative	West Indies	180	• Humanitarian • Societal	• wanting to help people • being respected and successful • fulfilling a sense of achievement.
13.	Why people apply to medical school: implications for widening participation activities.	McHarg J et al.	2007	Cross Sectional Qualitative	United Kingdom	15	• Scientific • Humanitarian	• desire to help people • desire for gainful employment • wish to give something to mankind • desire to save lives • to fulfil their lifelong ambition
14.	Comparison of Career Choice Motivation and Moral Reasoning Ability between Students in Baccalaureate and Graduate-entry Programs.	Kim MK	2007	Cross- Sectional Quantitative	Korea		• Scientific • Humanitarian • Societal	• Scientific interest • Opportunities to care for people • Status • Job security
15.	Demographics and motives of medical school applicants in Croatia.	Puljak L et al.	2007	Cross- Sectional Quantitative	Croatia	567	• Societal • Scientific	• Love for medical profession • Humanity of medicine • Interest in human body structure and function Interest in science • An opportunity to work/interact with people
16.	The attractions of medicine: the generic motivations of medical school applicants in relation to demography, personality and achievement	McManus IC et al.	2006	Cross Sectional Quantitative	London	2867	• Scientific • Humanitarian • Societal	• Indispensibility • Respect • Helping others • Science
Upper middle income countries								
17.	Achievement motivation level in students of Shiraz University of Medical Sciences and its influential factors.	Kavousipour S et al.	2015	Cross –Sectional Quantitative	Iran	770	• Societal	• family attitudes • getting good jobs in future • respect for themselves • the ability to learn • believing their role in victory and defeat • tendency toward optimism about themselves
18.	The characteristics of medical students and motivation towards career choice implications for curriculum	Korkmaz H et al.	2013	Cross- Sectional-mixed methods	Turkey	298	• Societal • Humanitarian	• Social status • Responsible job • High Income • Helping others
19.	Burnout and career choice motivation in medical students.	Pagnin D et al.	2013	Cross- Sectional Quantitative	Brazil	277	• Societal • Scientific • Humanitarian	• Intellectual curiosity • professional autonomy • altruism • interest in human relationships
20.	Factors Affecting Choice of Specialty Among First-year Medical Students of Four Universities in Different Regions of Turkey	Dikici FM et al.	2008	Cross- Sectional Quantitative	Turkey	717	• Societal • Scientific	• better financial opportunities • prestige • personal development • more benefits for the patient • wish to work in an urban area
Lower Middle Income Countries								
21.	Why become a doctor? Evaluation of motivational factors for selecting medical profession as a career	Kuriakose S	2015	Cross sectional Quantitative	India	100	• Humanitarian	• serve the poor and needy • own interest. • doctor in family. • near ones suffering from chronic illness or cancer, • themselves suffering from health problems • influenced by the lack of health care in rural areas.
22.	Indian medical students in public and private sector medical schools: are motivations and career aspirations different? – studies from Madhya Pradesh, India	Diwan V et al.	2013	Cross –Sectional Quantitative	India	792	• Humanitarian • Societal	• Personal choice • Parent’s wish • Service • Security • Prestige
23.	Why become a Doctor? Exploring the Career Aspirations and Apprehensions among Interns in South India	Seetharaman N et al.	2012	Cross- Sectional Quantitative	South India	147	• Humanitarian • Societal • Scientific	• Personal interest & passion for the profession • financial stability • parents’ wish
24.	Career Aspirations and Apprehensions Regarding Medical Education Among First Year Medical Students in Delhi	Lal P et al.	2007	Cross- Sectional Quantitative	India	189	• Humanitarian • Societal	• serve the sick and society • high status of a doctor in the society • father’s will • to earn money and • mother’s will

**Table 4:** Most commonly cited motivational factors among all the studies analyzed (*N* = 24)

	Motivational factors	Number of Sudies	Percentage of studies
Scientific factors			
1.	Interest in Medicine	16	66.7
2.	Professional growth/challenging career	6	25
3.	Work independence	3	12.5
4.	Work abroad/urban areas	3	12.5
Humanitarian factors			
5.	Work for people/help underprivileged	18	75
6.	Family experience of a disease/death	3	12.5
Societal factors			
7.	Social status/Prestige	8	33.3
8.	Financial security	6	25
9.	Job Security	5	20.8
10.	Parental wish	5	20.8
11.	Family Tradition	2	8.3

#### High-income countries

In most of the high-income counties, scientific and humanitarian factors were described as the main motivators to select medicine by medical students [15–29]. Most of the high income countries including Spain, Croatia, Poland, UK, Hungary, Germany and South Korea reported similar type of motivators to motivate the medical students for choosing medicine: interest in science/medicine, social interest, flexible work hours and work independence. Results reported by Kim et al.(2016) [16], Becker et al. (2015) [29], Wouters (2014) [19] emphasized on the scientific factors. Societal factors were also reported in most of these studies but fell lower in hierarchy.

#### Uppermiddle income countries

The main motivators to select medicine by medical students of upper-middle income countries include the societal and scientific factors [30–33]. A study by Kavousipour et al. (2015) [30] conducted in *Iran* explains that the factors which were most significant to motivate the students were family attitudes, getting good jobs in future, respect for themselves, the ability to learn, believing their role in victory and defeat and the tendency toward optimism about themselves. Pagnin et al. (2013) [32] also concluded similar findings. Social and professional status of the job, healthcare-people factor, others’ recommendation and advices, personal interest and nature of occupation, occupational experience and personal life had been identified as main factors of motivation. The findings reported by Korkmaz et al. (2013) [31] also found societal and scientific factors to be more significant motivators.

#### Lower-middle -income

In low-middle income countries, students have mixed responses for the choice of medical studies. [34–37]. Humanitarian and societal factors had been reported as main influences to join medicine.

Few studies conducted in various parts of India had reported almost similar results. A study conducted in Madhya Pradesh, India by Diwan et al. (2013) [35] concluded that reasons for entering medical education included personal ambition, parental desire, prestigious profession, altruistic reasons and pecuniary incentives. Similar to these findings were those reported by Kuriakose (2015) [34], Seetharaman et al. (2012) [36] and Lal et al. in 2007 [37]. The main reasons that motivate the medical students were to serve the sick and society and having a high status in society.

## Discussion

To our knowledge, this is the first systematic review of motivational factors for choosing medical studies by medical students globally. Earlier reviews were related to factors influencing student rating in undergraduate medical education course evaluations and factors that influence a career choice in primary care among medical students from high-, middle- and low-income countries [8]. The present systematic review, which has analyzed 24 studies in detail, is important as it identifies the motivational factors influencing the medical students to join medical studies in different parts of the globe along with the variations among the factors in lower-middle, upper-middle and high-income countries. As such, it provides essential insights into how students could be motivated, and how this varies across countries. No study was found from low-income countries. The limited research on this topic in low-income countries could be related to the lack of interest in this particular area, or to an overall deficit in research in developing nations, or both. These countries could identify the issues and intervene according to the research done in lower-middle and upper-middle income countries.

Several theories of motivation have been described in relation to career choice among student including intrinsic and extrinsic factors as described by Brissette and Howe [9] and by Maslow [38],. Taylor, McClelland and Herzberg [39]. However, Maslow’s theory remains to be the most detailed and frequently used theory [38]. The Maslow’s hierarchy of needs describes motivational factors under five broad segments: the physiological needs, the needs for safety and security, the needs for love and belonging, the needs for esteem, and the need to actualize the self, in that order [38]. Physiological needs are the basic needs required by an individual, such as food, water, sleep, etc. Once these needs are met, the second segment of needs comes into picture making safety, stability, protection the prime concerns. Following these factors the third segment consists of desires to marry, have a family, become a part of their community etc. The fourth segment of esteem has two versions as described by Maslow. The need for respect, prestige, prominence, magnificence, appreciation, attention, status, self-esteem, and dominance forms the lower version while the higher form involves the need for self-respect which includes feelings as self-confidence, capability, accomplishment, mastery, and freedom. The last segment is the phase of self-actualization which is a desire for self-fulfillment [38].

In low-middle income countries, students are still striving to fulfill primary basic needs and safety and security of employment, family, health. They fall under the first two segments of the pyramid comprising of basic needs, safety stability and protection and hence the predominant motivational factors are humanitarian in this group. In some areas where these needs are fulfilled, the higher segment of self-esteem also come into picture, hence societal factors are also seen in lower-middle income countries. The prime reasons for selecting medical studies among students in low-income countries were parental desire, respected profession and economic incentives, respect in society, high societal status and to serve the sick. The desire to serve the poor is deeply ingrained in this society. Most of the students belong to lower or middle socio-economic groups and understand the miseries of poor well and these factors lead them to serve the humanity and poor people. Here medical students are more sensitive to the social needs of population. The very reasons identified to take up medical career in these countries can be used to encourage students to take up medical studies. Mainly, the respect and feeling of altruism, followed by the monetary and social benefits are a driving force that can be used to attract the students into medical profession, hence improving the workforce. As the motivational factors are mostly innate, their further interest in medical studies and serving the nation will remain significant.

In the upper-middle income countries the factors as described by the middle zone in the Maslow’s hierarchy of needs pyramid were identified. The majority of studies identified societal factors as better predictors as compared to humanitarian and scientific factors. The main motivators to select medicine by medical students of upper-middle income countries are job security, social status, and parental wish. The reason behind this is that, to become a doctor is one of the highest ambition of many school-going students and their parents in middle- and low-income countries, along with the fact that the medical profession is preferred by the students due to its high prospect of financial security and high social status. Being a respected profession with high social status and higher salaries has been found to be motivating factor for students. The students in these countries have mostly met their basic needs and are more attracted towards a better lifestyle and income. Security in all fronts is a strong predictor for picking medical studies, and this can help enroll more students into this career. Excelling in their medical education may act as a strong target as their competition decides their future prospects.

The motivational factors commonly reported by most of the studies in high-income countries were the third and fourth segments of the Maslow’s hierarchy of needs pyramid. The scientific factors were the main motivators to select medicine by students. This may be due to the fact that the students in high-income countries chose medicine or science, who have prime interest in these subjects. The interest in science is usually developed during their school times to become medical school academics in a well-developed education system and with advanced technologies (modern laboratory facilities). The availability of good technologies and advanced education helps in developing specialized skills through the medical school years and beyond. In addition, the ability to earn well, pay their debts and live comfortably are strong motivators as well.

There are various strengths of the study. Firstly, the review was done on a sizeable number of 24 studies across the globe, hence generating stronger evidence. Secondly, the study relates the motivational factors across different countries with the Maslow’s hierarchy of needs theory [38]. This helps to understand the motivational factors of medical students to work in rural areas with respect to the innate motivational factors of a human being.

This review has a few limitations. Despite our efforts to identify all relevant studies by searching four different databases and using a fairly large number of search terms, we might have missed relevant studies. Additionally, unpublished studies from low- and middle-income countries were not represented (publication bias). The exclusion of articles published before 2006 may have omitted literature that could have provided valuable information. However, our review supplement two existing reviews published earlier [8, 9].

## Conclusion

In conclusion, this systematic review investigated the reasons that affect students’ decisions to join medical profession. The motivational factors are being classified in scientific factors (e.g. ‘interest in medicine’), societal factors (e.g. ‘respect/prestige’) and humanitarian factors (e.g.‘desire to help others’). The predominance of factors varied among students in high-, upper-middle and lower-middle income countries. Hence, this study offers cues to policy makers and educators in different countries to understand the motivational factors as a first step to formulate policy in order to tackle the shortage of health workers to improve the status of human resources across nations. However, more research on the subject would assist in promoting as well as translating health policy into concrete and effective measures at the local, national, regional and global levels in low- and middle- income countries.

## Abbreviations

MSMS: Motivation of selection of Medical Study
PRISMA: Preferred Reporting Items for Systematic Reviews and Meta-Analyses
WHO: World Health Organization
